# Scalable *In Situ* Hybridization on Tissue Arrays for Validation of Novel Cancer and Tissue-Specific Biomarkers

**DOI:** 10.1371/journal.pone.0032927

**Published:** 2012-03-08

**Authors:** Sara Kiflemariam, Sandra Andersson, Anna Asplund, Fredrik Pontén, Tobias Sjöblom

**Affiliations:** Department of Immunology, Genetics and Pathology, Science for Life Laboratory, Rudbeck Laboratory, Uppsala University, Uppsala, Sweden; The University of Kansas Medical Center, United States of America

## Abstract

Tissue localization of gene expression is increasingly important for accurate interpretation of large scale datasets from expression and mutational analyses. To this end, we have (1) developed a robust and scalable procedure for generation of mRNA hybridization probes, providing >95% first-pass success rate in probe generation to any human target gene and (2) adopted an automated staining procedure for analyses of formalin-fixed paraffin-embedded tissues and tissue microarrays. The *in situ* mRNA and protein expression patterns for genes with known as well as unknown tissue expression patterns were analyzed in normal and malignant tissues to assess procedure specificity and whether *in situ* hybridization can be used for validating novel antibodies. We demonstrate concordance between *in situ* transcript and protein expression patterns of the well-known pathology biomarkers *KRT17*, *CHGA*, *MKI67*, *PECAM1* and *VIL1*, and provide independent validation for novel antibodies to the biomarkers *BRD1*, *EZH2*, *JUP* and *SATB2*. The present study provides a foundation for comprehensive *in situ* gene set or transcriptome analyses of human normal and tumor tissues.

## Introduction

Precise and specific tissue localization of gene expression is instrumental for correct interpretation of transcriptome data from complex tissues such as patient tumors. The rapid accumulation of exome mutation data from tumors also yields novel putative therapeutic targets, and it is therefore valuable to investigate tissue gene expression to predict effects and side effects of novel cancer therapies. Specificity validation of diagnostic and therapeutic antibodies is another application where *in situ* gene expression analyses could contribute essential knowledge. To make optimal use of such *in situ* gene expression analyses in cancer biology, one needs (1) methods for facile and efficient generation of probes to any gene target, and (2) automated and robust procedures for staining of formalin-fixed paraffin-embedded (FFPE) tissues and tissue microarrays (TMAs).

In situ hybridization (ISH) techniques employ labeled RNA probes to analyze the expression and localization of specific mRNA transcripts in tissues at cell type resolution. Such probes are typically generated by *in vitro* transcription from plasmid or RT-PCR products in the presence of a hapten conjugated base such as digoxigenin-UTP. After hybridization, the bound probes are detected by chromogenic anti-hapten immunohistochemistry. Whereas frozen tissue sections are most frequently used in mRNA ISH, archival FFPE tissues and tissue microarrays can also be employed [1]. As mRNA ISH is technically difficult and encompasses many experimental steps, several different automation formats have been developed. The Tecan GenePaint system is an open robotic system that has successfully been used to chart the mouse transcriptome in frozen tissues [2], [3]. Briefly, the prehybridization, hybridization and signal detection reactions are performed in a flow-through chamber where an automated solvent system adds different solutions in parallel. This system together with the detection technology enables a daily throughput of up to two hundred slides [4]. Proteome-wide efforts have already demonstrated the feasibility of large scale FFPE immunostaining [5], [6]. Correlating protein and mRNA expression will provide an independent validation tool for both immunohistochemistry (IHC) and ISH, and enable the discovery of false positives of either method.

To obtain a flexible way to analyze tissue expression of large gene sets in FFPE tissue microarrays, we created a simple and scalable PCR-based procedure for generation of mRNA probes to any target gene in the same cDNA library and further developed an automated staining system, where 48 genes can be processed in 48 tissues or TMAs in a single run. We then validated the specificity and scalability of this approach by parallel ISH and IHC targeting well-known biomarkers in pathology. Finally, we demonstrate the specificity of antibodies to novel tissue-specific or cancer biomarkers using *in situ* hybridization.

## Results

### Technology optimization

Seventeen genes were chosen to represent well established pathology biomarkers or novel tissue-specific or cancer biomarkers discovered within the Human Protein Atlas project [5]. These included bromodomain containing 1 (*BRD1*), chromogranin-A (*CHGA*), histone-lysine N-methyltransferase (*EZH2*), family with sequence similarity 174 member B (*FAM174B*), glutamate decarboxylase 1 (*GAD1*), janus kinase 3 (*JAK3*), junction plakoglobin (*JUP*), keratin 17 (*KRT17*), v-yes-1 Yamaguchi sarcoma viral related oncogene homolog (*LYN*), mix1 homeobox-like 1 (*MIXL1*), antigen Ki-67 (*MKI67*), phosphodiesterase 6A (*PDE6A*), platelet endothelial cell adhesion molecule 1 (*PECAM1*), protein tyrosine phosphatase type C (*PTPRC*), special AT-rich sequence-binding protein 2 (*SATB2*), villin1 (*VIL1*) and zinc finger protein 473 (*ZNF473*). Desired PCR products and RNA probes were obtained for all genes (Figure S1).

We next determined whether RNA probe length affects sensitivity or specificity of ISH signals using two different approaches. In the first approach, RNA probes for *CHGA* and *KRT17* were fragmented in duplicate using alkaline- and metal ion-catalyzed methods to investigate if shorter probe length would give a higher signal due to better tissue penetration. Both protocols were adapted for use with DIG-UTP-labeled RNA probes. No significant difference in signal could be seen between fragmented and unfragmented probes when evaluating staining in normal colon, kidney, liver, spleen and tonsil (data not shown). The second approach was to investigate whether longer probes would have an impact on ISH signal. Three different probes targeting *PDGFRB* (platelet-derived growth factor receptor beta) with lengths of 500 bp, 1000 bp and 1500 bp were generated. No significant difference in signal between the probe lengths could be seen when evaluating staining in kidney glomeruli, however the background was increased for 1000 bp and 1500 bp probes when comparing sense and antisense RNA probes (data not shown) [7].

### Adoption of the GenePaint system for human tissue arrays

To facilitate a large-scale approach, optimization of proteinase K concentration was performed. Three genes with various expression levels, *PDGFRB, KRT17* and beta actin (*ActB*), were chosen and evaluated on a tissue microarray with normal colon, kidney, liver, spleen and tonsil. Concentrations tested were 2.5, 5, 10, 20, 40, 60, 80 and 100 µg/ml. For *ActB* a concentration of 40 µg/ml was sufficient to provide clear and distinct signals without affecting tissue morphology. For *KRT17* and *PDGFRB*, a proteinase K concentration of 60 µg/ml was optimal when comparing all the different tissues on the array and was therefore chosen for further experiments. To visualize genes expressed at low levels, double cycles of tyramide-based signal amplification was introduced. Furthermore, the importance of fresh tissue sections has been ascertained since weak or no signal was seen for tissue sections older than 3 weeks (data not shown).

### Validation of ISH staining on tissue arrays

**Figure 1:**
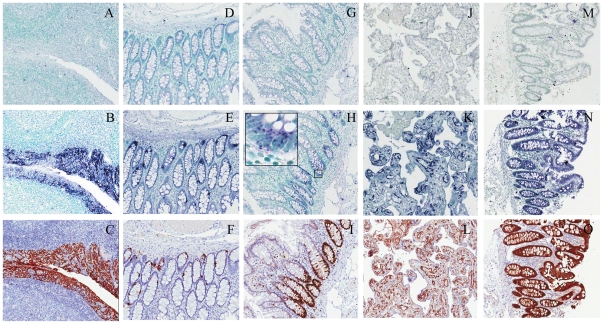
Validation of scalable *in situ* hybridization by parallel immunohistochemistry of pathology biomarkers on tissue arrays. ISH signals are seen as blue/purple staining with nuclei counterstained in methyl green, whereas IHC signals are in brown with hematoxylin counterstain. *A–C*: Keratin 17 (*KRT17*) in tonsil, *D–F*: Chromogranin A (*CHGA*) in colon, *G–I*: Ki-67 (*MKI67*) in colon, *J–L*: Platelet endothelial cell adhesion molecule 1 (*PECAM1*) in placenta, *M–O*: Villin-1 (*VIL1*) in colon. Tissue arrays were hybridized with sense control probes (A, D, G, J, M), antisense probes (B, E, H, K, N), or immunostained with antibodies (C, F, I, L, O) targeting the respective transcripts or protein products. All images were derived from slides scanned with a 40× objective.

Consecutive tissue sections were stained using sense probe ISH, antisense probe ISH and immunohistochemistry to compare the gene expression on the mRNA and protein level. For a subset of the analyzed genes (*CHGA*, *KRT17*, *LYN*, *MKI67*, *PDE6A*, *PECAM1*, *PTPRC* and *VIL1*), immunohistochemistry-based protein expression patterns are well known and established. These targets were used to compare the expression of protein and the corresponding transcripts for validation of the scalable ISH procedure. The intermediate filament protein KRT17 was, as expected, highly expressed in a wide selection of differentiated epithelial cells, as demonstrated by staining of the tonsil (Figure 1, A–C and Figure S3) and in bronchus (Figure S3). CHGA, a marker for neuroendocrine differentiation, showed strong staining in neuroendocrine cells of the gut (Figure 1, D–F), in pancreatic islets and endocrine organs such as the parathyroid (Figure S3). The well characterized proliferation marker Ki-67 is expressed in G1, S, G2 and M stages of the cell cycle and often used to assess proliferating cells within tumors. Proliferating cells in the base of normal colonic crypts (Figure 1, G–I), in anal vulva and in tonsil (Figure S3) are shown. The ISH signal for Ki-67 was localized to specific regions in the nucleus in agreement with data from the mouse [8]. PECAM1 was expressed in the endothelial cells in most organs, exemplified by the richly vascularized placenta (Figure 1, J–L), endometrium (pre menopause) and lymph node (Figure S3). VIL1 is a protein expressed in the brush border of epithelia and as such highly expressed in kidney tubules and glandular cells of the gastrointestinal tract, such as the colon (Figure 1, M–O), appendix and duodenum (Figure S3). Furthermore, for *CHGA*, *PECAM1* and *VIL1*, two independent RNA probe pairs (Table S1) were used for cross validation of probe specificity. The tissue and cellular distribution pattern of expression for both mRNA and protein were similar for these five genes, confirming the sensitivity and specificity of automated in situ hybridization on FFPE TMAs. For PDE6A, IHC showed staining in alveoli, skeletal muscle, bronchus and cells in the renal glomeruli. mRNA and protein correlation was only seen in two cases of skeletal muscle. Established antibodies for LYN and PTPRC showed distinct membranous and cytoplasmic staining in lymphoid tissues and glandular cells of the gastrointestinal tract, and selective cytoplasmic staining in lymphoid tissue, respectively. No correlation was seen between protein and mRNA expression (Figure S5). For *PTPRC*, the lack of ISH staining was cross validated with two independent RNA probe pairs (Table S1).

### Validation of novel antibody biomarkers by ISH on tissue arrays

**Figure 2:**
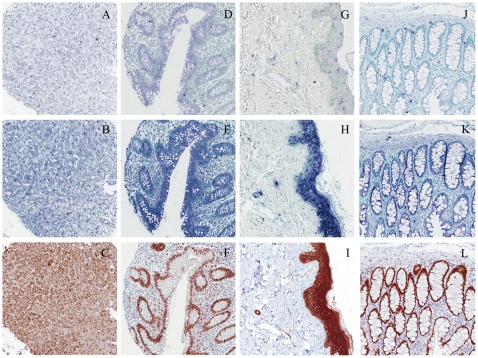
Validation of potential tissue-specific and cancer antibody biomarkers by in situ hybridization on tissue arrays. ISH signals are seen as blue/purple staining with nuclei counterstained in methyl green, whereas IHC signals are in brown with hematoxylin counterstain. *A–C*: Bromodomain containing 1 (*BRD1*) in adrenal gland, *D–F*: Histone-lysine N-methyltransferase (*EZH2*) in appendix (nuclear and cytoplasmic staining for protein and mRNA, respectively), *G–I*: Junction plakoglobin (*JUP*) in skin, *J–L*: Special AT-rich sequence-binding protein 2 (*SATB2*) in colon (nuclear and cytoplasmic staining for protein and mRNA, respectively). Tissue arrays were hybridized with sense control probes (A, D, G, J), antisense probes (B, E, H, K), or immunostained with antibodies (C, F, I, L) targeting the respective transcripts or protein products. All images were derived from slides scanned with a 40× objective.

The antibody-derived expression in the Human Protein Atlas project suggested either a tissue-specific expression or clinical usefulness in cancer diagnostics or therapy for the novel putative biomarkers *BRD1*, *EZH2*, *FAM174B*, *GAD1*, *JAK3*, *JUP*, *MIXL1*, *SATB2* and *ZNF473*. Therefore, analysis of their *in situ* mRNA and protein expression in normal tissues could provide validation of antibody specificity. BRD1 is known by immunohistochemistry to be expressed in Purkinje and neuronal cells in CNS, cells in seminiferus ducts in testis, glandular cells in prostate, adrenal gland and appendix, and macrophages in lung, which was confirmed by ISH (Figure 2, A–C and Figure S4). EZH2 is a potential cancer biomarker as overexpression of the nuclear protein is seen in a variety of aggressive cancers, including breast cancer, prostate cancer and glioblastoma multiforme [9]. Gene expression was found on both transcript (cytoplasmic staining) and protein (nuclear staining) in glandular cells in appendix (Figure 2, D–F), duodenum and fallopian tube (Figure S4), cells in seminiferus ducts in testis and myocytes in heart muscles. JUP was suggested by IHC to be a tissue-specific biomarker expressed in adnexal and epidermal cells of the skin, which was confirmed by ISH (Figure 2, G–I) and in squamous epithelial cells of the tonsil (Figure S4). SATB2 is a novel biomarker for colorectal cancer [10], where gene expression profiling has demonstrated association of transcriptional downregulation with metastasis and poor prognosis [11]. Antibodies to SATB2 showed nuclear staining in glandular cells of the lower gastrointestinal tract, including colonic crypt cells (Figure 2, L). Concomitant array ISH confirmed SATB2 transcript expression in cytoplasm restricted to the epithelium of the appendix, colon (Figure 2, J–K) and rectum (Figure S4), but not other organs. For validation of these four novel biomarkers, two independent RNA probe pairs were used for each gene (Table S1). GAD1 showed similar ISH and IHC staining in Purkinje cells and cells in the molecular layer of the cerebellum, but no correlation was seen in other tissues. The specificity of the novel antibodies to *FAM174B*, *JAK3*, *MIXL1* and *ZNF473* could not be verified by ISH (Figure S5). For *JAK3*, the lack of ISH staining was cross validated with two independent RNA probe pairs but for *FAM174B*, *MIXL1* and *ZNF473*, only one probe pair could be designed due to either too short protein coding regions or too high homology with other genes (Table S1).

### Parallel analyses of SATB2 protein and transcript expression on FFPE colorectal cancer arrays

**Figure 3:**
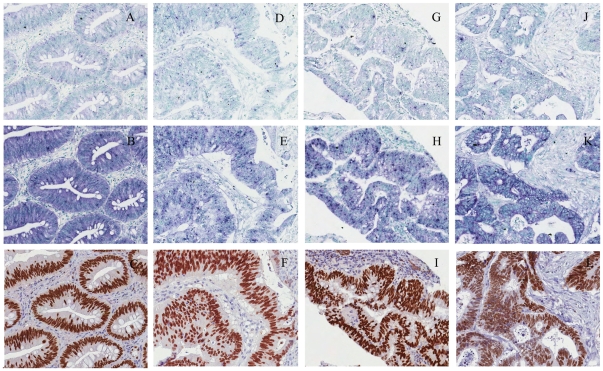
Validation of SATB2 antibody by in situ hybridization in a selection of arrayed colorectal cancers. ISH signals are seen as blue/purple staining with nuclei counterstained in methyl green, whereas IHC signals are in brown with hematoxylin counterstain. Tissue arrays were hybridized with sense control probe (A, D, G, J), antisense probe (B, E, H, K) or immunostained with antibody (C, F, I, L). All images were derived from slides scanned with a 40× objective.

Protein and mRNA expression of SATB2 was assessed in colorectal cancers using an array encompassing 60 primary tumors in duplicate. Annotation was performed based on a three-grade scale. One pair did not contain tumor tissue, and of the remaining 59 samples, 44 showed complete concordance between IHC and ISH expression in glandular cells (Figure 3). Twelve showed semi concordance as staining was observed but with different intensities, whereas 3 samples displayed no correlation. Agreement between protein and mRNA expression pattern was observed in a total of 56 samples (Cohen's kappa test, κ = 0.68).

## Discussion

Scaling ISH approaches to gene set or exome-wide analyses requires pipelines for probe synthesis, hybridization, analysis and annotation. Whereas much ground work has been performed in the framework of large mouse transcriptome studies on frozen tissues, modifications are necessary for successful and reproducible ISH in FFPE materials. For facile probe synthesis, we conclude that careful informatics combined with the use of a pooled human cDNA library (MegaMan) entails a high first-pass success rate in probe template generation. This cDNA library encompasses mRNA from 32 human tissues and 34 human cancer cell lines ensures a good representation of transcripts with diverse expression levels and splice variants. In parallel projects, this approach has successfully been used to generate a total of 700 probes with a first-pass success rate of >95% (data not shown).Essential factors for successful staining that differ from previously published procedures employing frozen tissues were the use of freshly sectioned tissue, since weak or no staining was observed for older FFPE tissue sections possibly due to oxidation of RNA on the exposed surface or unsatisfactory fixation conditions affecting RNA quality, a higher concentration of proteinase K, and two cycles of tyramide biotin-based signal amplification. Although the latter may result in slightly higher background levels, it is in our experience a convenient way to increase signal intensity.

The specificity and sensitivity of ISH was thoroughly evaluated by staining of consecutive sections from the same TMAs encompassing ∼40 normal tissue types in the human body. The ISH and IHC staining patterns of *KRT17*, *CHGA*, *MKI67*, *PECAM1* and *VIL1* were identical across tissue types, a selection of representative samples are shown in Figure 1 and Figure S3, thus providing strong validation of the ISH approach. This high degree of concordance is in agreement with previous observations from mouse transcriptome projects. However, a much needed improvement to enable efficient data mining of ISH and IHC is a standardized ontology for annotation of human tissues, in analogy to the EMAP mouse anatomy ontology [3].

Antibody sensitivity and specificity is a major concern in immunohistochemistry, especially in large scale projects where numerous antibodies are being produced towards targets with unknown expression patterns. Specificity validation of antibodies to diagnostic grade may be performed by obtaining highly similar staining pattern with an antibody raised to a different epitope of the same antigen [6], loss of signal in knock-out mice or other model organism, or highly similar staining pattern with *in situ* hybridization. We here demonstrate the feasibility of the latter approach at organism scale in the human, as TMAs encompassing virtually all normal tissues can be analyzed in one single experiment. We were able to verify the specificity of novel antibodies to *BRD1*, *EZH2*, *JUP* and *SATB2*, with two independent RNA probe pairs, confirming that ISH can provide independent specificity validation. A selection of representative samples is seen in Figure 2 and Figure S4. In parallel experiments, we were unable to confirm specificity of the novel antibodies to *FAM174B*, *JAK3*, *MIXL1* and *ZNF473* (Figure S5). The semi-correlation between mRNA and protein expression for *GAD1* and *PDE6A* can be due to either antibody specificity or differences in transcriptional and translational processes. The lack of correlation between mRNA and protein expression for *LYN* and *PTPRC* can be due to biological and technical reasons. The antibodies for *LYN* and *PTPRC* are targeting all known isoforms of the proteins and the RNA probe pairs are located in regions which are present in all known transcripts. Also for PTPRC, two independent RNA probe pairs were used, demonstrating lack of ISH staining between inter TMA replicates. We therefore believe that the lack of correlation between mRNA and protein expression is most likely due to biological reasons, such as post-transcriptional, translational and post-translational modifications affecting the levels of mRNA and protein. Also, mRNA decay and protein half-life could have an impact on mRNA and protein correlation. Other sources of discrepancy between IHC and ISH could be lack of sensitivity or specificity in either one of the approaches; this enables interpretation of concordant staining patterns as supportive of antibody specificity and discordant patterns as lack of specificity. In large scale efforts, antibodies with concordant ISH staining patterns should be prioritized for further analyses as opposed to antibodies with discordant or lacking ISH staining.

*SATB2*, a nuclear matrix-associated transcription factor and a member of the family of special AT-rich binding proteins, has recently been shown to be expressed in normal cells of the lower gastrointestinal tract and in cancer cells of colorectal origin. Due to the highly specific nuclear expression pattern in normal and malignant cells of the gastrointestinal tract, SATB2 protein expression was suggested as a clinically useful diagnostic biomarker for colorectal cancers, the third most commonly diagnosed cancer in the world [10]. Cohen's Kappa test was performed to determine the agreement between IHC and ISH staining, demonstrating good inter-rater reliability. The scalable technology presented here also enables gene set expression analyses in human tissue arrays. We envision that the tyrosine kinome, tyrosine phosphatome, other genes in cancer pathways, along with a multitude of novel candidate cancer genes derived from exome and genome sequencing constitute prime targets for large scale *in situ* hybridization based characterization. We propose that comprehensive and systematic mapping of the expression *in situ* in normal and malignant human tissues at cell type resolution will provide valuable knowledge, especially in cancer drug development as the results can aid in predicting effects, guide repositioning of available targeted drugs, and help predict response to drugs that inhibit several different molecular targets. It also provides the technical foundation for transcript expression mapping of human protein-encoding genes, and potentially also other RNA species, in systematic whole-genome approaches.

## Materials and Methods

### Ethics statement

The use of HPA tissue arrays in this study is covered by the HPA ethical permit (EPN Uppsala 2002/577, 2005/338) and ethical permit to the investigators (EPN Uppsala 2007/116). The identities of arrayed tissue samples are not known to the investigators, nor will they be release into the public domain.

### Probe generation

The probe generation procedure is outlined in Figure S2. We developed an in-house software that selects transcript regions suitable for hybridization probe design in the genes of interest by minimizing homology with transcripts of other genes in the human RefSeq transcriptome, ensuring that the probe sequences are present in RefSeq transcripts of the gene of interest, and including regions to span exon boundaries. Two independent PCR primer sets for amplification of 500–600 nt products were generated for each gene using Primer3 [12]. A T7 promoter sequence (5′-GCGTAATACGACTCACTATAGGG-3′) was incorporated in the 5′-end of the forward or reverse primer, respectively, to generate the sense or antisense riboprobe template. All primer pairs used in probe generation are summarized in Table S1.

Stratagene's MegaMan human transcriptome library, a collection of cDNA created using mRNA from 32 human tissues and 34 human cancer cell lines, was used for PCR (www.genomics.agilent.com). Each reaction contained 2 µl 10×PCR buffer (166 mM (NH_4_)_2_SO_4_, 670 mM Tris pH 8.8, 100 mM 2-mercaptoethanol, 67 mM MgCl_2_), 2 µl dNTPs (10 mM, GE Healthcare, Uppsala, Sweden), 1.2 µl DMSO (Sigma-Aldrich, St. Louis, MO, USA), 0.4 µl forward primer (50 µM, Sigma-Aldrich), 0.4 µl reverse primer (50 µM, Sigma-Aldrich), 0.2 µl Taq polymerase (5 U/µl, Invitrogen, Carlsbad, CA, USA), 1 µl MegaMan human transcriptome library (160 ng/µl, (Agilent Technologies, Santa Clara, CA, USA)) and MilliQ water to a volume of 20 µl. The touchdown PCR conditions included annealing temperatures ranging from 64°C to 57°C; 96°C for 2 min; 3 cycles of 96°C for 10 s, 64°C for 10 s and 70°C for 30 s; 3 cycles of 96°C for 10 s, 61°C for 10 s and 70°C for 30 s; 3 cycles of 96°C for 10 s, 58°C for 10 s and 70°C for 30 s; 41 cycles of 96°C for 10 s, 57°C for 10 s and 70°C for 30 s, and a final extension for 5 min at 70°C. The PCR products were run on a 1.25% agarose gel for product size confirmation. Sense and antisense riboprobes labeled with digoxigenin were generated by *in vitro* transcription of PCR products with DIG RNA labeling mix (Roche, Rotkreuz, Switzerland) in a total reaction volume of 5 µl. One µg of each RNA probe was run on a 6% TBE-Urea gel (Invitrogen) for size confirmation. Stocks were made by diluting RNA probes with MilliQ water to 200 ng/µl and stored in −80°C. Probe working solutions of 20 ng/µl were made from stock solutions and stored in −20°C.

RNA probes were fragmented using two different approaches. For alkaline-catalyzed fragmentation, giving fragments ranging from 50–150 bp, RNA probes were digested with 0.2 M sodium carbonate buffer (pH 10.2) at 60°C. The incubation time is calculated by t = L_0_−L_f_/*k*L_0_L_f_, where t is incubation time in minutes, L_0_ and L_f_ are initial and final lengths of the probe in kb, and *k* is the rate constant for hydrolysis (approximately 0.11 kb^−1^min^−1^). The reactions were stopped by adding sodium acetate (pH 4.7) and samples were ethanol precipitated [13]. One µg of each RNA probe was run on a 6% TBE-Urea gel for size confirmation. For metal ion-catalyzed fragmentation, giving fragments between 60–200 bp, RNA probes were incubated with 100 mM zinc chloride in 100 mM Tris-HCl (pH 7) at 70°C for 15 min. The reactions were stopped with 0.5 M EDTA (pH 8) [14]. One µg of each RNA probe was run on a 6% TBE-Urea gel for size confirmation.

### Tissue preparation

The tissue arrays used were the standard arrays utilized for immunohistochemistry in the Human Protein Atlas project (www.proteinatlas.org). These arrays encompass triplicate 1-mm cores of 48 different types of non-malignant human tissue [15] and duplicate 1-mm cores of primary colorectal cancer [10]. Tissue microarrays with FFPE tumor and normal tissues were sectioned to 6 µm and mounted on Superfrost Plus microscope slides.

### In situ hybridization

Tissue sections were dewaxed in xylene, followed by hydration in a graded ethanol series (100%, 95% and 80%, 3 min in each). The hybridization was automated using Tecan GenePaint (Tecan Ag, Männedorf, Switzerland) essentially as described [2], [4], [16]. All procedures were performed at room temperature unless otherwise stated. Briefly, endogenous peroxidase activity was blocked in 0.7% H_2_O_2_ in methanol, followed by deproteinization in 0.2 M HCl and digestion by 60 µg/ml proteinase K (Roche). The tissue sections were pre-hybridized in hybridization buffer (Ambion, Foster City, CA, USA) without probe for 30 min and then incubated with 200 ng/ml digoxigenin-labeled riboprobe for 4 hours at 64°C. After hybridization, tissue sections were washed in 5× saline-sodium citrate (SSC), 50% formamide and 0.1× SSC. To detect bound probe, an anti-digoxigenin antibody (150 U/ml, Roche) conjugated to horseradish peroxidase was added. After five washes in blocking buffer, a tyramide biotin amplification step was performed to increase the in situ detection sensitivity (Perkin Elmer, Waltham, MA, USA). The deposited biotin was detected by alkaline phosphatase-conjugated neutravidin (2 mg/ml, ThermoScientific, Waltham, MA, USA), which cleaves the chromogenic substrate nitroblue tetrazolium (NBT) 5-bromo-4-chloro-3-indolyl phosphate (BCIP) to produce a blue/purple precipitate at the site of hybridization. After 30 min development time, the chromogenic reaction was stopped by incubating the tissue sections in a buffer containing EDTA, followed by fixation in 4% PFA. Nuclei were counterstained with 2% methyl green and the slides were dehydrated in graded alcohols and mounted in a resin-based medium.

### Immunohistochemistry

Briefly, slides were deparaffinized in xylene, hydrated in graded alcohols and blocked for endogenous peroxidase in 0.3% hydrogen peroxide diluted in 95% ethanol. For antigen retrieval, a decloaking chamber (Biocare Medical, Walnut Creek, CA; USA) was used. Slides were immersed and boiled in citrate buffer, pH6 (Lab Vision, Värmdö, Sweden) for 4 min at 125°C and then allowed to cool to 90°C. Automated IHC was performed essentially as described [17], using an Autostainer 480 instrument (Lab Vision). Tissue sections were incubated with primary antibodies (Table S2) and a dextran polymer visualization system (UltraVision LP HRP polymer, Lab Vision) for 30 min each at room temperature and slides were developed for 10 min using diaminobenzidine (Lab Vision) as chromogen. All incubations were followed by a rinse in wash buffer (Lab Vision). Slides were counterstained in Mayer's hematoxylin (Histolab, Gothenburg, Sweden) and cover slipped using Pertex (Histolab) as mounting medium. Incubation with 1× PBS instead of primary antibody served as negative control.

### Annotation of ISH and IHC tissues

The Aperio ScanScope CS Slide Scanner system (Aperio Technologies, Vista, CA, USA) was used to digitize whole-slide images of ISH and IHC arrays at 40-fold magnification. The intensity of ISH and IHC staining was manually evaluated and scored using an annotation system similar to that described in [18] with a three grade scale; where 0 denotes absence of signal, 1 presence of weak to moderate signal and 2 presence of strong signal. Cohen's Kappa test was used to determine the agreement between ISH and IHC.

## Supporting Information

Figure S1 **Generation of PCR product and RNA probes.** (**A**) PCR products (200 ng/well) were separated on a 1.25% agarose gel. The expected band sizes were observed in all cases. Lanes 1 and 21; 1 kb DNA ladder, lanes 20 and 38; negative controls, lanes 2 and 3; sense and antisense for BRD1 (566 bp), lanes 4 and 5; CHGA (546 bp), lanes 6 and 7; EZH2 (534 bp), lanes 8 and 9; JUP (517 bp), lanes 10 and 11; KRT17 (510 bp), lanes 12 and 13; MKI67 (533 bp), lanes 14 and 15; PECAM1 (528 bp), lanes 16 and 17; SATB2 (505 bp), lanes 18 and 19; VIL1 (514 bp), lanes 22 and 23; FAM174B (228 bp), lanes 24 and 25; Gad1,(401 bp), lanes 26 and 27; JAK3 (505 bp), lanes 28 and 29; LYN (510 bp), lanes 30 and 31; MIXL1 (402 bp), lanes 32 and 33; PDE6A (568 bp), lanes 34 and 35; PTPRC (555 bp), and lanes 36 and 37; ZNF473 (516 bp). (**B**) RNA probes (1 µg/well) were separated on a 6% TBE-Urea gel. The expected band sizes were observed in all genes. Lanes 1, 14, 22 and 34; 0.1–2 kb RNA ladder, lanes 13 and 40; negative controls, lanes 2 through 39; same loading scheme as DNA gel in (A).
(TIF)

Figure S2 **Probe generation procedure.** Schematic representation of the procedure.
(TIF)

Figure S3 **Representative samples of concordant data for the validation genes.** ISH signals are seen as blue/purple staining with nuclei counterstained in methyl green, whereas IHC signals are in brown with hematoxylin counterstain. Keratin 17 (*KRT17*) in bronchus (left panel) and cervix, uterine (right panel); Chromogranin A (*CHGA*) in pancreas (left panel) and parathyroid gland (right panel) using an independent RNA probe pair; Ki-67 (*MKI67*) in anal vulva (left panel) and tonsil (right panel); Platelet endothelial cell adhesion molecule 1 (*PECAM1*) in endometrium, pre menopause (left panel) and lymph node (right panel) using an independent RNA probe pair; Villin-1 (*VIL1*) in appendix (left panel) and duodenum (right panel) using an independent RNA probe pair. All images were derived from slides scanned with a 40× objective.
(TIF)

Figure S4 **Representative samples of concordant data for the novel tissue or cancer specific biomarkers.** ISH signals are seen as blue/purple staining with nuclei counterstained in methyl green, whereas IHC signals are in brown with hematoxylin counterstain. Bromodomain containing 1 (*BRD1*) in lung (left panel) and prostate (right panel); Histone-lysine N-methyltransferase (*EZH2*) in duodenum (left panel, nuclear and cytoplasmic staining for protein and mRNA, respectively) and fallopian tube (right panel, nuclear and cytoplasmic staining for protein and mRNA, respectively); Junction plakoglobin (*JUP*) in two different tonsil specimens (left and right panel) within the same TMA; Special AT-rich sequence-binding protein 2 (*SATB2*) in two different rectum specimens (left and right panel, nuclear and cytoplasmic staining for protein and mRNA, respectively) within the same TMA. All ISH staining were cross validated with an independent RNA probe pair. All images were derived from slides scanned with a 40× objective.
(TIF)

Figure S5 **Representative samples of concordant and discordant data.** ISH signals are seen as blue/purple staining with nuclei counterstained in methyl green, whereas IHC signals are in brown with hematoxylin counterstain. Glutamate decarboxylase 1 (*GAD1*) in two different cerebellum specimens (left and right panel) within the same TMA; Phosphodiesterase 6A (*PDE6A*) in two different skeletal muscle specimens (left and right panel) within the same TMA; Family with sequence similarity 174 member B (*FAM174B*) in endometrium, postmenopause (left panel) and stomach (right panel); Janus kinase 3 (*JAK3*) in epididymis (left panel) and breast (right panel) with two independent RNA probe pairs; v-yes-1 Yamaguchi sarcoma viral related oncogene homolog (*LYN*) in appendix (left panel) and rectum (right panel); Mix1 homeobox-like 1 (*MIXL1*) in breast (left panel) and seminal vesicle (right panel); Protein tyrosine phosphatase type C (*PTPRC*) in lymph node with two independent RNA probe pairs (left panel) and in spleen with two independent RNA probe pairs (right panel); Zinc finger protein 473 (*ZNF473*) in rectum (left panel) and tonsil (right panel). All images were derived from slides scanned with a 40× objective.
(TIF)

Table S1 **PCR primer sequences used for RNA probe generation.**
(XLS)

Table S2 **Primary antibodies used for immunohistochemistry.**
(XLS)
