# Supplementary figures and images for: Scalable In Situ Hybridization on Tissue Arrays for Validation of Novel Cancer and Tissue-Specific Biomarkers
Source: PLoS One. 2012 Mar 8;7(3):e32927. doi: 10.1371/journal.pone.0032927 (PMC3297615; doi:10.1371/journal.pone.0032927)

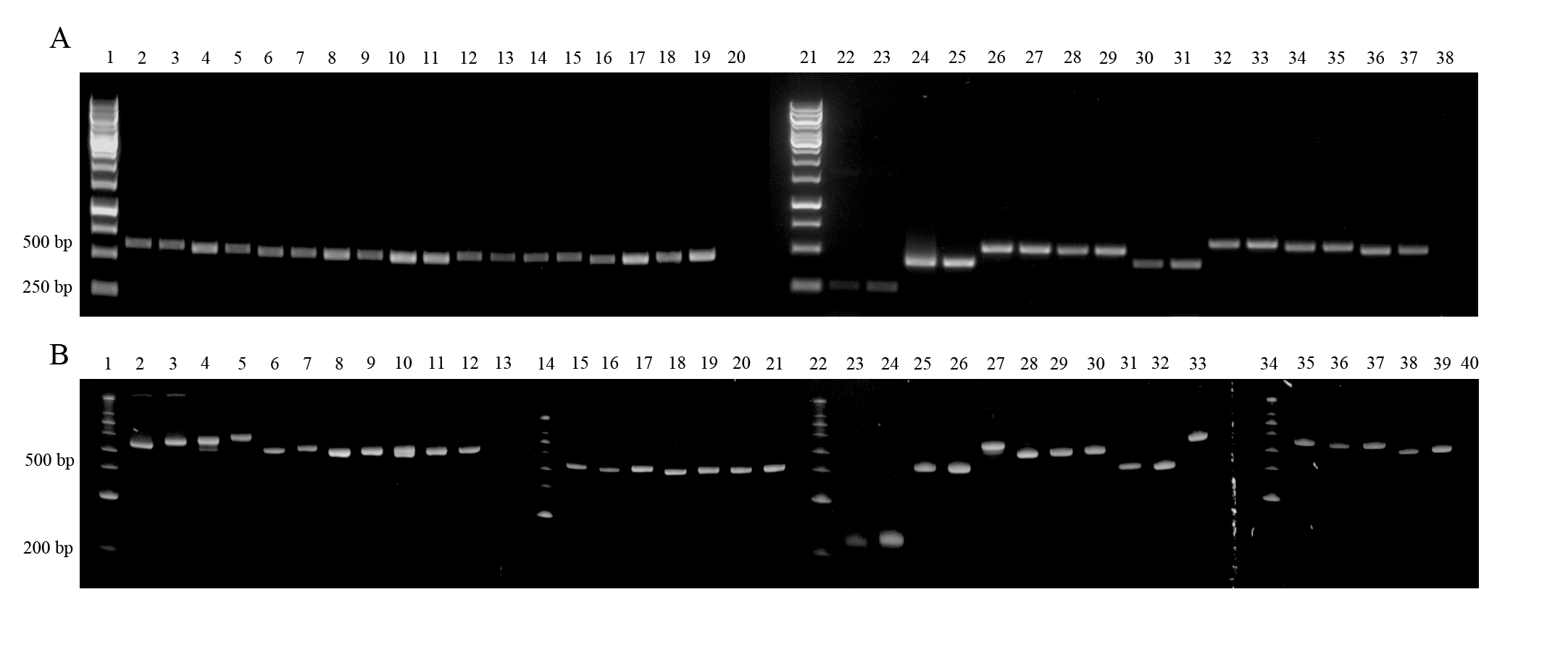

Supplement: Figure S1 — Generation of PCR product and RNA probes. (A) PCR products (200 ng/well) were separated on a 1.25% agarose gel. The expected band sizes were observed in all cases. Lanes 1 and 21; 1 kb DNA ladder, lanes 20 and 38; negative controls, lanes 2 and 3; sense and antisense for BRD1 (566 bp), lanes 4 and 5; CHGA (546 bp), lanes 6 and 7; EZH2 (534 bp), lanes 8 and 9; JUP (517 bp), lanes 10 and 11; KRT17 (510 bp), lanes 12 and 13; MKI67 (533 bp), lanes 14 and 15; PECAM1 (528 bp), lanes 16 and 17; SATB2 (505 bp), lanes 18 and 19; VIL1 (514 bp), lanes 22 and 23; FAM174B (228 bp), lanes 24 and 25; Gad1,(401 bp), lanes 26 and 27; JAK3 (505 bp), lanes 28 and 29; LYN (510 bp), lanes 30 and 31; MIXL1 (402 bp), lanes 32 and 33; PDE6A (568 bp), lanes 34 and 35; PTPRC (555 bp), and lanes 36 and 37; ZNF473 (516 bp). (B) RNA probes (1 µg/well) were separated on a 6% TBE-Urea gel. The expected band sizes were observed in all genes. Lanes 1, 14, 22 and 34; 0.1–2 kb RNA ladder, lanes 13 and 40; negative controls, lanes 2 through 39; same loading scheme as DNA gel in (A). (TIF) [file pone.0032927.s001.tif]

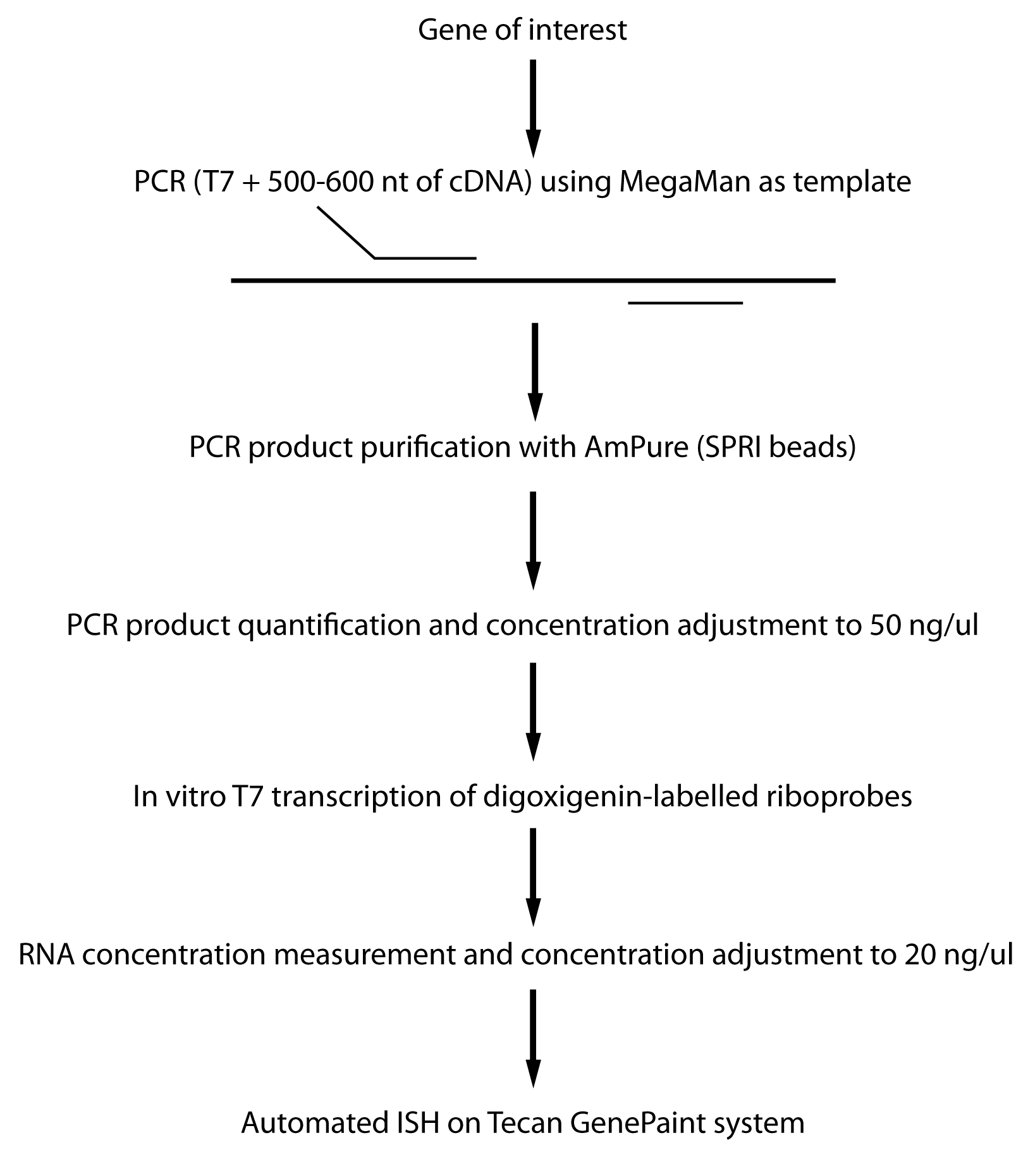

Supplement: Figure S2 — Probe generation procedure. Schematic representation of the procedure. (TIF) [file pone.0032927.s002.tif]

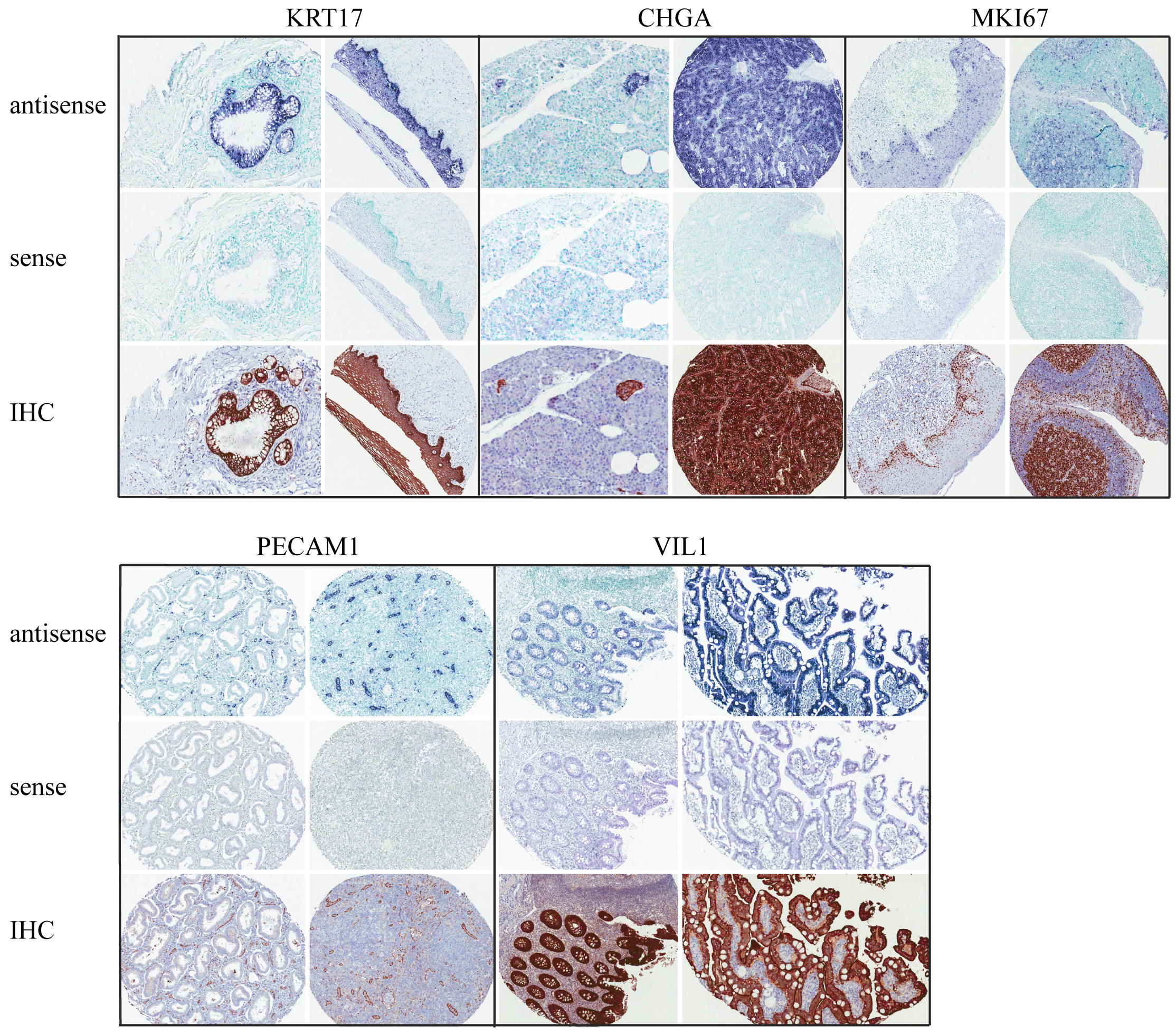

Supplement: Figure S3 — Representative samples of concordant data for the validation genes. ISH signals are seen as blue/purple staining with nuclei counterstained in methyl green, whereas IHC signals are in brown with hematoxylin counterstain. Keratin 17 (KRT17) in bronchus (left panel) and cervix, uterine (right panel); Chromogranin A (CHGA) in pancreas (left panel) and parathyroid gland (right panel) using an independent RNA probe pair; Ki-67 (MKI67) in anal vulva (left panel) and tonsil (right panel); Platelet endothelial cell adhesion molecule 1 (PECAM1) in endometrium, pre menopause (left panel) and lymph node (right panel) using an independent RNA probe pair; Villin-1 (VIL1) in appendix (left panel) and duodenum (right panel) using an independent RNA probe pair. All images were derived from slides scanned with a 40× objective. (TIF) [file pone.0032927.s003.tif]

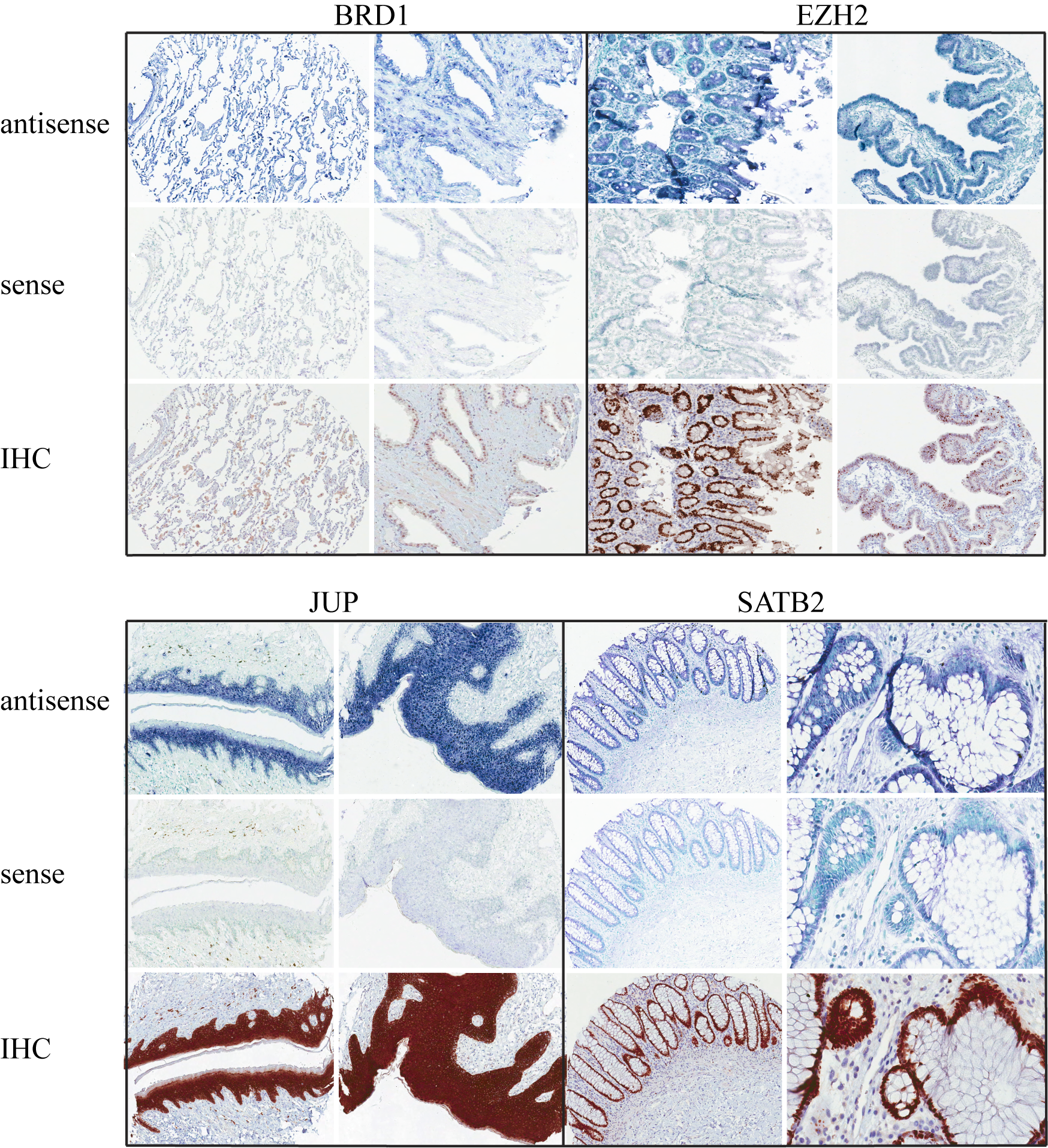

Supplement: Figure S4 — Representative samples of concordant data for the novel tissue or cancer specific biomarkers. ISH signals are seen as blue/purple staining with nuclei counterstained in methyl green, whereas IHC signals are in brown with hematoxylin counterstain. Bromodomain containing 1 (BRD1) in lung (left panel) and prostate (right panel); Histone-lysine N-methyltransferase (EZH2) in duodenum (left panel, nuclear and cytoplasmic staining for protein and mRNA, respectively) and fallopian tube (right panel, nuclear and cytoplasmic staining for protein and mRNA, respectively); Junction plakoglobin (JUP) in two different tonsil specimens (left and right panel) within the same TMA; Special AT-rich sequence-binding protein 2 (SATB2) in two different rectum specimens (left and right panel, nuclear and cytoplasmic staining for protein and mRNA, respectively) within the same TMA. All ISH staining were cross validated with an independent RNA probe pair. All images were derived from slides scanned with a 40× objective. (TIF) [file pone.0032927.s004.tif]

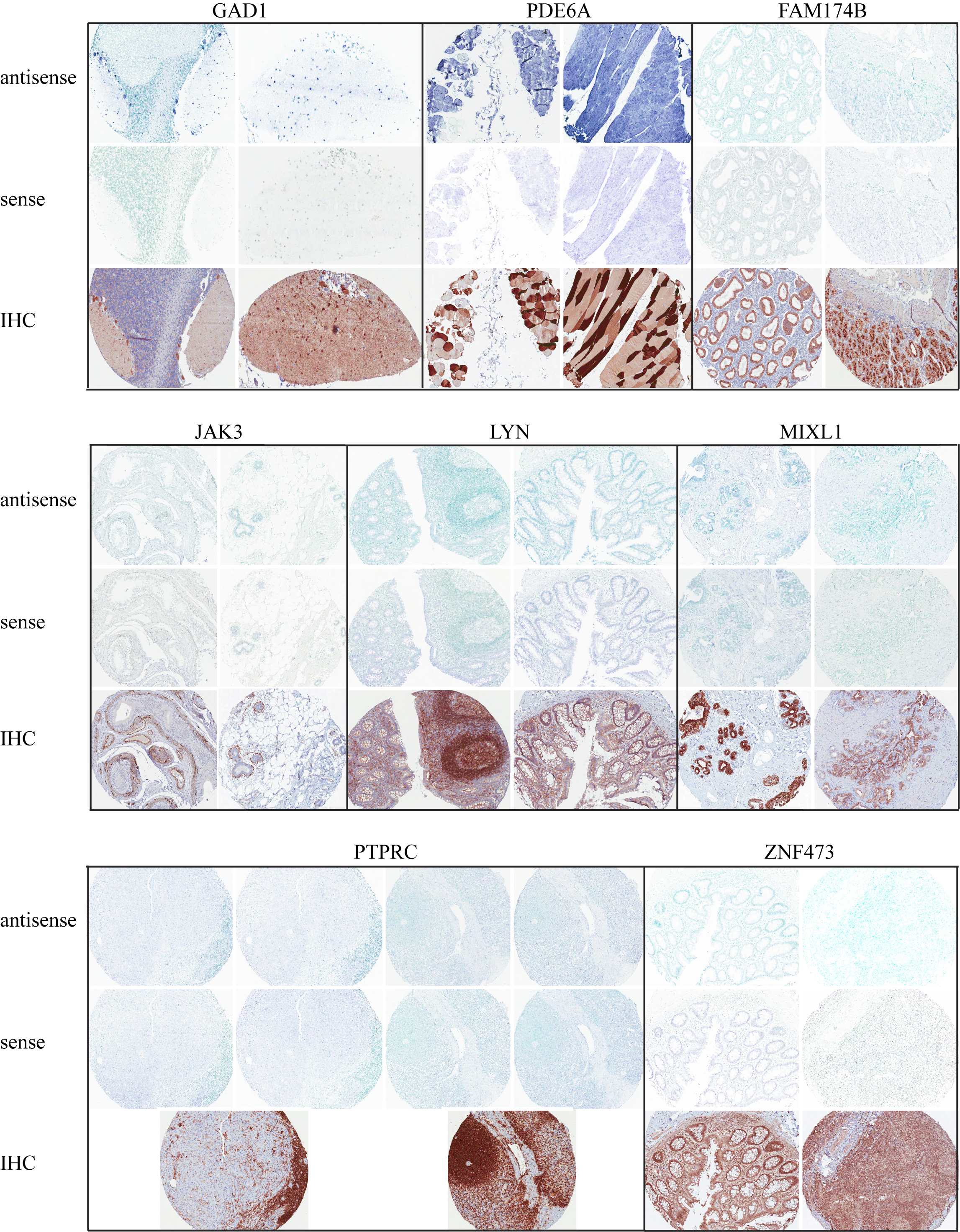

Supplement: Figure S5 — Representative samples of concordant and discordant data. ISH signals are seen as blue/purple staining with nuclei counterstained in methyl green, whereas IHC signals are in brown with hematoxylin counterstain. Glutamate decarboxylase 1 (GAD1) in two different cerebellum specimens (left and right panel) within the same TMA; Phosphodiesterase 6A (PDE6A) in two different skeletal muscle specimens (left and right panel) within the same TMA; Family with sequence similarity 174 member B (FAM174B) in endometrium, postmenopause (left panel) and stomach (right panel); Janus kinase 3 (JAK3) in epididymis (left panel) and breast (right panel) with two independent RNA probe pairs; v-yes-1 Yamaguchi sarcoma viral related oncogene homolog (LYN) in appendix (left panel) and rectum (right panel); Mix1 homeobox-like 1 (MIXL1) in breast (left panel) and seminal vesicle (right panel); Protein tyrosine phosphatase type C (PTPRC) in lymph node with two independent RNA probe pairs (left panel) and in spleen with two independent RNA probe pairs (right panel); Zinc finger protein 473 (ZNF473) in rectum (left panel) and tonsil (right panel). All images were derived from slides scanned with a 40× objective. (TIF) [file pone.0032927.s005.tif]
